# Cellular adaptive immune response against porcine circovirus type 2 in subclinically infected pigs

**DOI:** 10.1186/1746-6148-5-45

**Published:** 2009-12-22

**Authors:** Esther Steiner, Carole Balmelli, Heidi Gerber, Artur Summerfield, Kenneth McCullough

**Affiliations:** 1 Institute of Virology and Immunoprophylaxis, Sensemattstrasse 293, 3147 Mittelhäusern, Switzerland; 2 Dr. Esther Steiner, Theodor Kocher Institute, University of Bern, Freiestrasse 1, CH 3012 Bern, Switzerland; 3 Dr. Carole Balmelli, Agroscope Changins-Wädenswil Research Station ACW, 1260 Nyon 1, Switzerland

## Abstract

**Background:**

Porcine circovirus type 2 (PCV2) is a dominant causative agent of postweaning multisystemic wasting syndrome (PMWS), a multifactorial disease complex with putative immunosuppressive characteristics. Little is known about adaptive PCV2-specific immune responses in infected pigs. Therefore, the T and B cell responses following PCV2 infection in 3-week old SPF piglets infected with PCV2 or PCV2 plus porcine parvovirus (PPV) were studied.

**Results:**

All animals were asymptomatically infected. At 7 days post infection (d p.i.), B lymphocyte and T lymphocyte numbers decreased in the dual infected, but not in the single infected piglets. At this time point a transient PCV2 viraemia was noted in the PCV2 infected groups. Antibodies against the infecting virus were detectable at day 24-28 p.i. for anti-PCV2 antibodies and at day 10 p.i. for anti-PPV antibodies, with no apparent influence of PCV2 on the early PPV antibody development. In the animals infected with PPV alone, IFN-γ secreting cells (SC) that were not specific for PCV2 were detected by ELISPOT assay at day 7 p.i. Interestingly, this response was absent in the PCV2/PPV dual infected animals. PCV2-specific IFN-γ SC were observed in the PCV2/PPV infected group at 7 d p.i. and in the PCV2 single infected group at 21 d p.i. A reduction in the numbers of IFN-γ SC was observed following anti-CD4 and anti-CD8 antibody treatment, suggesting roles for both CD4^+ ^and CD8^+ ^T cells in the response against PCV2 infection. This was supported by an observed increase in the percentage of IFN-γ positive CD8^hi ^cytotoxic T cells as well as IFN-γ positive CD8^-/low ^helper T cells after PCV2 *in vitro *re-stimulation.

**Conclusions:**

Infection of weaned SPF piglets with PCV2 alone or combined with PPV does not induce disease and in both cases a relatively slow anti-PCV2 antibody response and weak T lymphocyte responses were found. Knowledge on such immunological characteristics is important for both PCV2 pathogenesis and vaccination.

## Background

Porcine circovirus type 2 (PCV2) is a non-enveloped, circular single stranded DNA virus belonging to the Circoviridiae virus family [1], and is the causative agent of a number of diseases in swine, particularly postweaning multisystemic wasting syndrome (PMWS) [2]. The genome of PCV2 is 1759 nucleotides long, making it one of the smallest viruses replicating autonomously in mammalian cells [3]. PCV2 was first isolated from tissues of PMWS diseased pigs in Canada [4], the US and Europe [5] in 1998. Disease has been reproduced by viral co-infection of colostrum-deprived or gnotobiotic piglets with PCV2 and porcine parvovirus (PPV) [6,7]. Particular vaccine adjuvant administration has also been shown to assist development of PMWS disease after experimental PCV2 infection [8,9]. Nevertheless, PCV2 is clearly the primary causative agent of PMWS [10], requiring the secondary factors such as co-infections or vaccinations for full expression of the disease. It is assumed that secondary viral infections or the administration of immunostimulatory compounds activate PCV2 infected cells to divide [9], thus promoting the replicative cycle of PCV2, which is dependent on the host DNA polymerase [11].

Nevertheless, neither natural nor experimental PCV2 infection together with cofactors will induce disease in all infected animals. On the farm, PCV2-induced diseases have been found to increase the pig mortality rate from 2-3% to 14-30%. PMWS-diseased animals are most often in the age range of 8-12 weeks old. They display clinical symptoms of wasting, diarrhoea, jaundice, respiratory distress and enlarged lymph nodes [12]. Typical histological findings are also reported in lymph nodes: The follicular architecture is lost, lymphocytes are depleted, histiocytes and multinucleated giant cells infiltrate the lymph nodes, and basophilic inclusion bodies are detected in the histiocytes [13]. In addition, interstitial pneumonia, mononuclear inflammatory infiltration in the liver, lymphoplasmacytic colitis and peri-endarteritis are observed [14,15].

The induction of anti-PCV2 neutralizing antibodies (Ab) was shown to correlate with protection from disease [16]. While important, Ab are effective at targeting extracellular virus and cell surface antigen (Ag) only. PCV2 has been shown to infect epithelial, endothelial and monocytic cells *in vivo *[14,15,17,18], and *in vitro *observations on primary cells have confirmed that this reflects replicating virus [19-22]. Considering that PCV2 is a non-enveloped virus, its capsid protein is unlikely to be expressed at the surface of infected cells; indeed, there is no evidence that this occurs. Accordingly, virion proteins could not represent a target for Ab-mediated immune defence against PCV2-infected cells; other cytotoxic mechanisms would be required. While natural killer cells and the cytotoxic T lymphocyte (CTLs)-based immune defences are strong candidates for eradicating PCV2-infected cells, there have been no studies characterizing the existence of PCV2-specific T cell responses - neither CTLs nor T helper (Th) cell responses. Such immune parameters are particularly important for asymptomatic animals, and therefore for our understanding of immune defences in animals resisting disease development.

One study of the immune compartments influenced by PCV2 infection demonstrated a lymphopenia involving naïve CD4^+^CD8^- ^Th cells, memory/effector CD4^+^CD8^+ ^Th cells and CD4^-^CD8^+ ^CTLs in diseased, but not healthy animals [23]. Interleukin 10 (IL-10) and interferon gamma (IFN-γ) have also been noted following *in vitro *PCV2 re-stimulation [24], suggesting the existence of both regulatory and stimulatory/effector pathways. In contrast, there has been no study elaborating on the development of T lymphocyte responses in asymptomatic, PCV2-infected animals. Therefore, the present study was conducted to analyse and characterise the PCV2-specific T cell responses developing during experimental PCV2 and PCV2/PPV dual infection in asymptomatic animals.

## Results

### Clinical monitoring and daily weight gain

**Figure 1:**
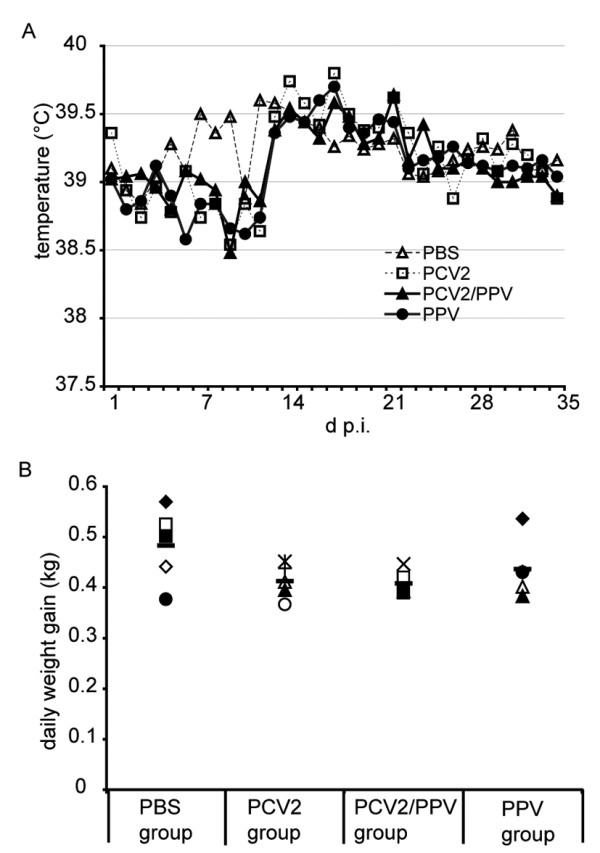
**Body temperature and average daily weight gain of animals infected with PCV2 and/or PPV**. (A) Body temperature. The rectal body temperature was measured for all animals at daily intervals. Mean values of the groups are shown. (B) Weight gain. The average daily weight gain (kg) is shown for each animals within a group (filled and open symbols); group mean values are represented by the bar symbol.

Following intranasal infection of the 3-week old SPF piglets with PCV2, PPV or PCV2/PPV, as described in Materials and Methods, enlarged inguinal and popliteal lymph nodes were noted in the PPV single and PCV2/PPV dual infected animals from day 10 to day 17 p.i. This was taken to indicate the presence of the PPV infection. None of the animals in any group displayed fever during the 35 days of experiment (Fig. 1A); a slight decrease in temperature was noted for the PCV2 single, PPV single and PCV2/PPV dual infected animals compared with the PBS group until day 13 p.i. (Fig. 1A). This was considered to be influenced by differences in food management until day 13 p.i., because the PBS control animals had remained in the SPF breeding unit while the infected animals were in the containment facility. The ad libitum food management can also explain the higher average daily weight gain in the PBS group (Fig. 1B). Overall, the average daily weight gain did not significantly differ among the PCV2/PPV dual, PCV2 single and PPV single infected animal groups (Fig. 1B).

### PPV specific antibodies are present from day 10 p.i. in the PPV infected animals

**Figure 2:**
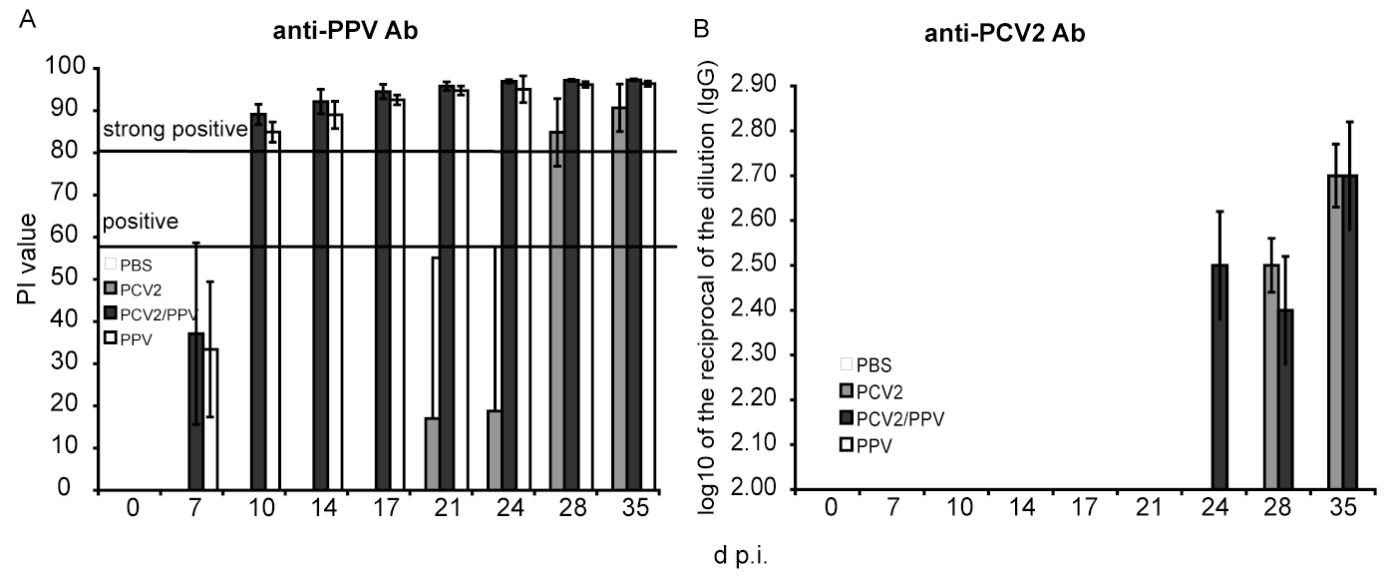
**Anti-PPV and anti-PCV2 Ab titres**. (A) Anti-PPV specific antibodies were measured with a commercial competitive Ab ELISA, at time points (days p.i.) indicated on the x-axis. The values are given as percent inhibition (PI), calculated as described in Materials and Methods. Means of the groups, +/- SD, are shown.(B) Anti-PCV2 IgG Ab titres were measured by an indirect ELISA. The log_10 _of the reciprocal end-point dilution is given for the time points (days p.i.) indicated on the x-axis. Mean values for the groups are shown by the histograms, +/- standard deviation (SD).

The presence of anti-PPV Ab was analysed by a commercial competitive Ab ELISA. At day 10 p.i., all PCV2/PPV dual and PPV single infected animals had seroconverted against PPV (Fig. 2A). The percentage inhibition (PI) values reached 84% and 89% respectively (Fig. 2A), increasing to 94% and 95% at day 21 p.i., and remaining at these levels during the remainder of the observation period of 35 days (Fig. 2A). While the animals of the PBS group were always negative for anti-PPV Ab, the animals of the PCV2 single infected group seroconverted at day 28 p.i. against PPV (Fig. 2A). This may be explained by the fact that the PCV2 single infected animals were handled by the same persons in the same building as the PPV infected animals, even though the animals were in separate rooms with separate ventilation; in contrast, the PBS control group was housed and handled separately in an SPF unit. The PPV contamination of the PCV2 single infected group happened at around day 10-14 p.i. Therefore, this group was excluded from all statistical comparisons after day 10 p.i.

### Analysis of anti-PCV2 antibodies and PCV2 viraemia

When assayed for anti-PCV2 specific IgG, the PBS control group values were always below background level. The plasma samples from the PPV single infected animals also remained below background levels. Seroconversion against PCV2 was clearly observed at day 24 p.i. in the PCV2/PPV dual infected group, and 4 days later in the PCV2 single infected group (Fig.2B). At day 28 p.i., all of the PCV2 infected animals had seroconverted against PCV2; the Ab titres increased to comparable levels at day 35 p.i. in both groups (Fig. 2B).

The presence of PCV2 DNA in the piglet plasma was analysed by TaqMan real time PCR to seek any evidence of PCV2 viraemia. Samples from the PBS control and the PPV single infected animals all tested negative for the presence of PCV2 DNA (data not shown). In contrast, PCV2 DNA was detected in plasma samples from four of the PCV2 single and all five of the PCV2/PPV dual infected animals at day 7 p.i. (average Ct value +/- SD of 38.9 +/- 5 and of 38.6 +/- 3.5 respectively). Interestingly, this apparent viraemia was transient; all other time points tested gave negative results (data not shown).

Further analysis of PCV2 viraemia was attempted in terms of the presence of viral antigen in PBMC. Intracellular staining for the viral Capsid (Cap) protein by flow cytometry failed to detect antigen, neither in CD172a(SWC3)^+ ^monocytes nor in CD4^+ ^lymphocytes (data not shown).

### PCV2/PPV dual infected animals display a transient decrease in leukocyte and lymphocyte counts

The total leukocytes were counted for individual animals in each group, and the group values compared statistically. A significant decrease in the leukocyte levels was detected in the PCV2 single and PCV2/PPV dual infected groups between days 7 and 10 p.i. (43 and 46 × 10^6 ^cells/ml respectively) compared with the PBS controls (data not shown). Interestingly, the PPV group showed the highest levels of leukocytes at this time (79 × 10^6^cell/ml) (data not shown).

**Figure 3:**
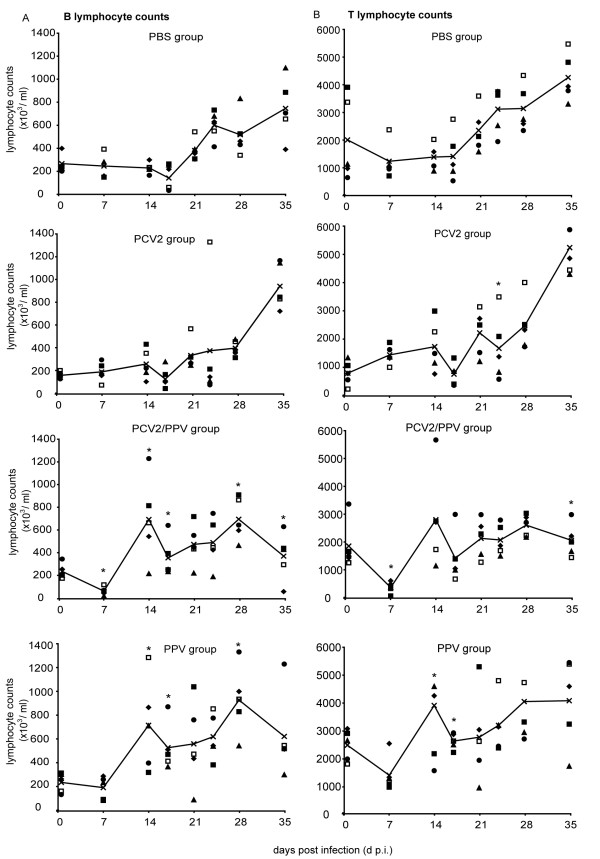
**B- and T-lymphocyte counts**. (A) B lymphocyte counts. PBMC were isolated at the time points (days post-infection (p.i.)) indicated on the x-axis, and stained with anti-IgG H+L Ab to analyse the B cell numbers. The absolute B cell counts were calculated and expressed as ×10^3 ^cells/ml. Individual animals are represented by the open and filled symbols, with the mean values for the groups shown by the continuous line. (B) T lymphocyte counts. The above PBMC were stained for CD3a expression, to analyse the T lymphocyte counts. These counts are given as × 10^3 ^cells/ml for the individual animals (open and filled symbols), with the group mean values shown as the continuous line. *P < 0.05.

In order to analyse the leukocytes in more detail, and to relate to previous publications, the B- and T-lymphocyte numbers were determined by staining for surface IgG and CD3a. Consistent with the observed decrease in the leukocyte numbers, the B cell counts in the PCV2/PPV dual infected group declined significantly to all other groups at 7 d p.i. (Fig 3A). However, this was not observed with the PCV2 single infected group. Interestingly, a comparable significant rise in B cell levels was noted, in the PCV2/PPV dual and PPV single infected groups, at day 14 p.i., compared to the PBS group. (Fig. 3A). The significantly higher B cell numbers persisted in the PPV single infected group until day 35, compared to the PBS group. The B cell number in the PCV2/PPV dual infected animals was significantly lower than the B cell number in the PPV and PBS group at day 35 p.i. (Fig. 3A).

A significant decrease was also observed for the T lymphocytes at day 7 p.i. in the PCV2/PPV dual infected group, but not in the other groups (Fig. 3B). While an increase was noted in this group at day 14 p.i., this was statistically significant from the PBS control group only for the PPV single infected group (Fig. 3B). Again similar to the B cell counts, the T cell numbers significantly decreased at day 35 p.i. in the PCV2/PPV dual infected animals compared to the PPV single infected group or PBS group (Fig. 3B).

Overall, the data shows a decrease in leukocyte and lymphocyte numbers in the PCV2/PPV dual infected animals, even though the animals remained clinically asymptomatic. Although there was a "recovery" of the leukocyte numbers thereafter, this did not continue to increase with the PCV2/PPV dual infected animals in the same fashion as in the PBS control group and PPV infected group. This was particularly the case for the T lymphocytes.

### PBMC of PCV2 infected animals respond to PCV2 re-stimulation by IFN-γ secretion

**Figure 4:**
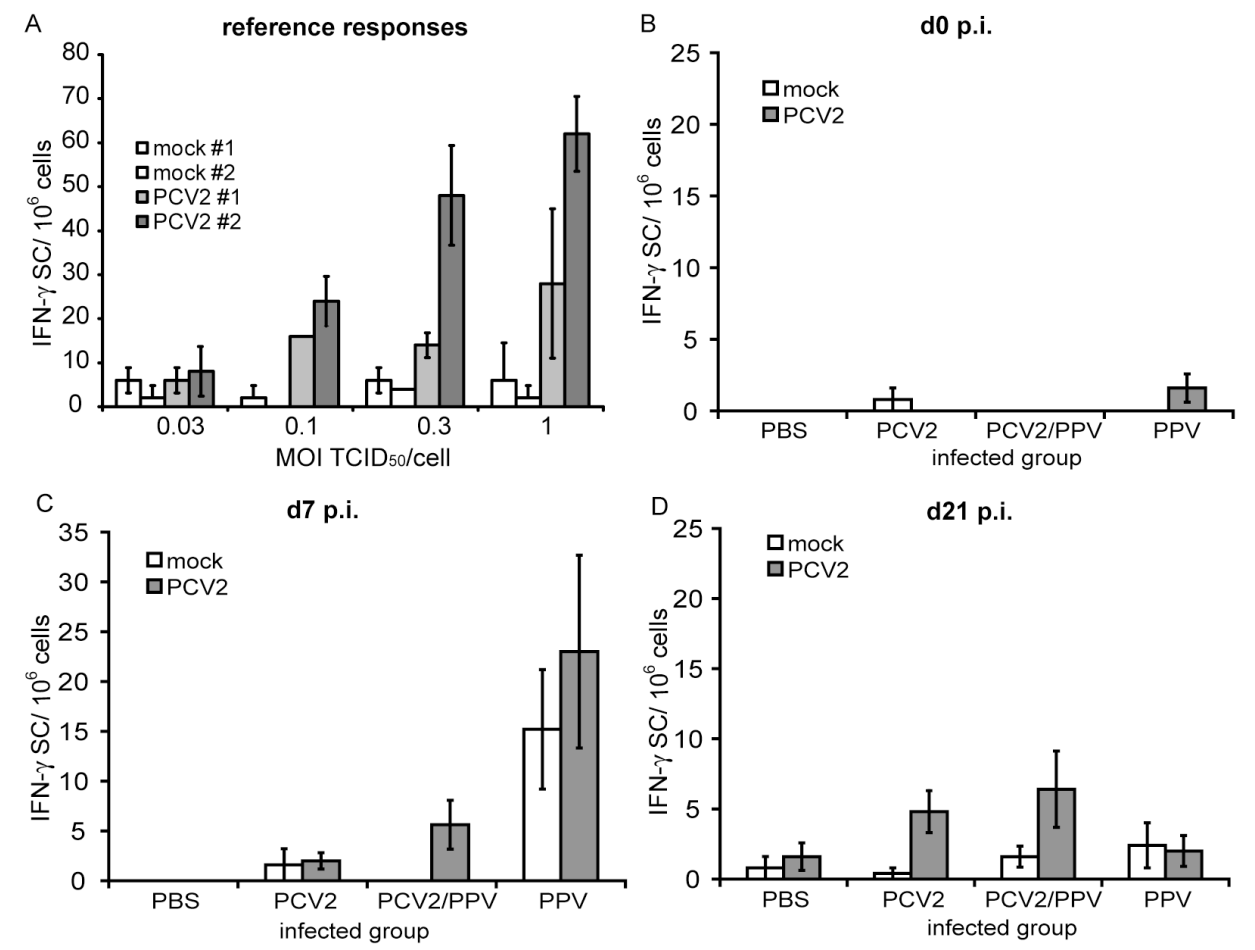
**IFN-γ secreting cells after *ex vivo *PCV2 re-stimulation**. (A) PBMC from two adult PCV2-immune animals were used as positive controls to evaluate the ability of the test to detect PCV2 specific IFN-γSC. The PBMC were re-stimulated for 24 h with PCV2-infected cell lysate, or mock cell lysate as negative control, at increasing MOI based on the titre of the PCV2 (TCID_50_/cell, x-axis). The IFN-γSC were measured by ELISPOT assay as described in Materials and Methods, and expressed per 10^6 ^cells. (B-D) PBMC were isolated from the piglets which had received PBS (control), PCV2 single infection, PCV2/PPV double infection and PPV single infection. The cells were re-stimulated with PCV2 or mock antigen at 1 TCID_50_/cell (or equivalents dilution for the mock) for 24 h, and the IFN-γSC detected by ELISPOT assay. The mean values of the groups, +/- SD, are shown for PBMC isolated before infection (0 days) (B), 7 d p.i. (C) and 21 d p.i. (D). The IFN-γSC are calculated per 10^6 ^cells.

IFN-γ ELISPOT assay for *ex-vivo* PCV2 re-stimulation of PBMC was employed to quantify the PCV2 specific IFN-γSC arising following infection. Firstly, PBMC were freshly isolated from two PCV2 immune adult pigs, to determine the levels and range of IFN-γSC which could be expected. The cells were re-stimulated with increasing amounts of PCV2 or mock for 24 h. A clear PCV2-specific response was observed in terms of the numbers of IFN-γSC/10^6 ^cells; this increased in a dose-dependent manner when the re-stimulation employed PCV2 (Fig. 4A).

The above permitted an analysis of the time-dependent appearance of PCV2-specific IFN-γSC following PCV2 infection, using freshly isolated PBMC from the PBS control, PCV2 single, PPV single or PCV2/PPV dual infected piglets. The number of IFN-γ SC/10^6 ^cells at day 0 remained at background levels for all groups (Fig. 4B). In three of the five PCV2/PPV dual infected animals, PCV2 specific IFN-γ SC were detected at day 7 p.i. (Fig. 4C). This was clearly PCV2 specific, because re-stimulation with mock did not lead to IFN-γ secretion. In contrast, the number of IFN-γ SC in the PCV2 single infected animals was similar to the background levels obtained with mock antigen stimulation. By day 21 p.i., PBMC from both PCV2/PPV dual and PCV2 single infected groups gave a PCV2-specific IFN-γ SC response upon *in vitro *re-stimulation (Fig. 4D). In both groups, four out of five animals responded to the virus re-stimulation, giving IFN-γ SC numbers similar to or slightly higher than the numbers obtained for the PBMC from the PCV2/PPV dual infected group at day 7 p.i.

An interesting result was observed with the PBMC from the PPV single-infected group. At day 7 p.i., the level of IFN-γ SC raised in the PPV single infected group regardless if cells were restimulated with PCV2 or mock antigens (Fig. 4C). Although this observed response was the highest for all the groups, it was not PCV2-specific. Moreover, the level of activation did not relate to that obtained with cells from the PCV2/PPV dual infected group (Fig. 4C) and the response against the mock antigen was not observed when the animal donors of the PBMC had been dually infected with PCV2 and PPV (Fig. 4C). These results suggested that an unspecific IFN-γ response is elicited in PBMC from the PPV single infected animals and is absent in the PCV2/PPV animals, which only display an anti-PCV2 specific response.

By day 21 p.i., PBMC from the PPV single-infected group were still capable of responding similarly to both PCV2 and mock antigens, but now the response was lower than that observed at day 7 p.i. (Fig. 4D). Moreover, the IFN-γ SC response was lower than the PCV2-specific response obtained with PBMC from the PCV2 infected groups (single and dual) stimulated with PCV2 (Fig. 4D).

Overall, it can be seen that the PCV2/PPV dual and the PCV2 single infected groups did generate PCV2-specific lymphocytes, in terms of IFN-γ SC. These were clearly identifiable at 7 and 21 days p.i., respectively. There was variation in the number of IFN-γ SC/10^6 ^cells, both between the groups and between animals, ranging from 4-16 IFN-γ SC/10^6 ^cells between individual piglets responding in a positive and PCV2-specific manner.

### Contribution of CD4^+ ^and CD8^+ ^cells in the PCV2-specific recall response

**Figure 5:**
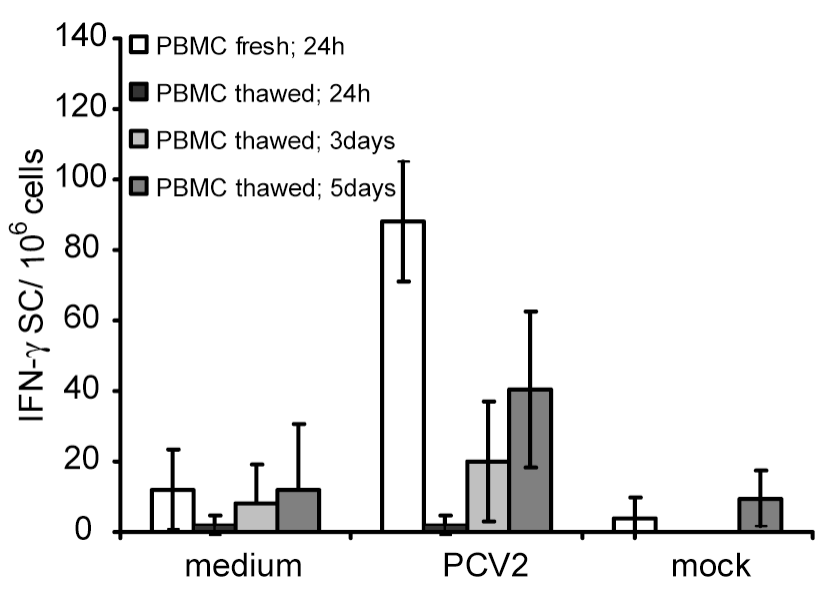
**Comparison of the PCV2 re-stimulation profile for freshly isolated compared to frozen and then *in vitro *expanded PBMC**. PBMC were re-stimulated with PCV2, mock antigen, or medium alone directly after isolation ("PBMC fresh; 24 h"). Aliquots of the PBMC were frozen under liquid nitrogen, before thawing and expanding by culture in the presence of rpoIL-2 together with PCV2, mock antigen, or medium alone. This expansion was for 24 h ("PBMC thawed; 24 h"), 3 days ("PBMC thawed; 3 days") or 5 days ("PBMC thawed; 5 days") prior to analysis for IFN-γ SC by the ELISPOT assay. Means of triplicates +/- SD of two experiments are shown.

In order to characterize the nature of the IFN-γ SC detected by the above *ex vivo* analyses, the recall response of frozen PBMC samples from the PCV2-immune piglets was analysed in the presence of anti-CD4 and anti-CD8 Ab [25]. The thawed PBMC were expanded for 5 days in the presence of the virus plus 50 U/ml of rpoIL-2, to expand antigen-specific T-cells expressing higher levels of CD25 (IL-2 receptor α chain) than unspecific naive cells [26]. An initial experiment was performed to ascertain if PBMC which had been frozen would retain their capacity to respond against the PCV2 antigen. Fig. 5 shows that the frozen PBMC efficiently responded to the PCV2 re-stimulation in terms of IFN-γ SC, albeit with a lower frequency of IFN-γ SC compared with freshly isolated cells. Moreover, the detection sensitivity for the IFN-γ SC assay was increased when the re-stimulated PBMC were cultured for 5 days.

**Figure 6:**
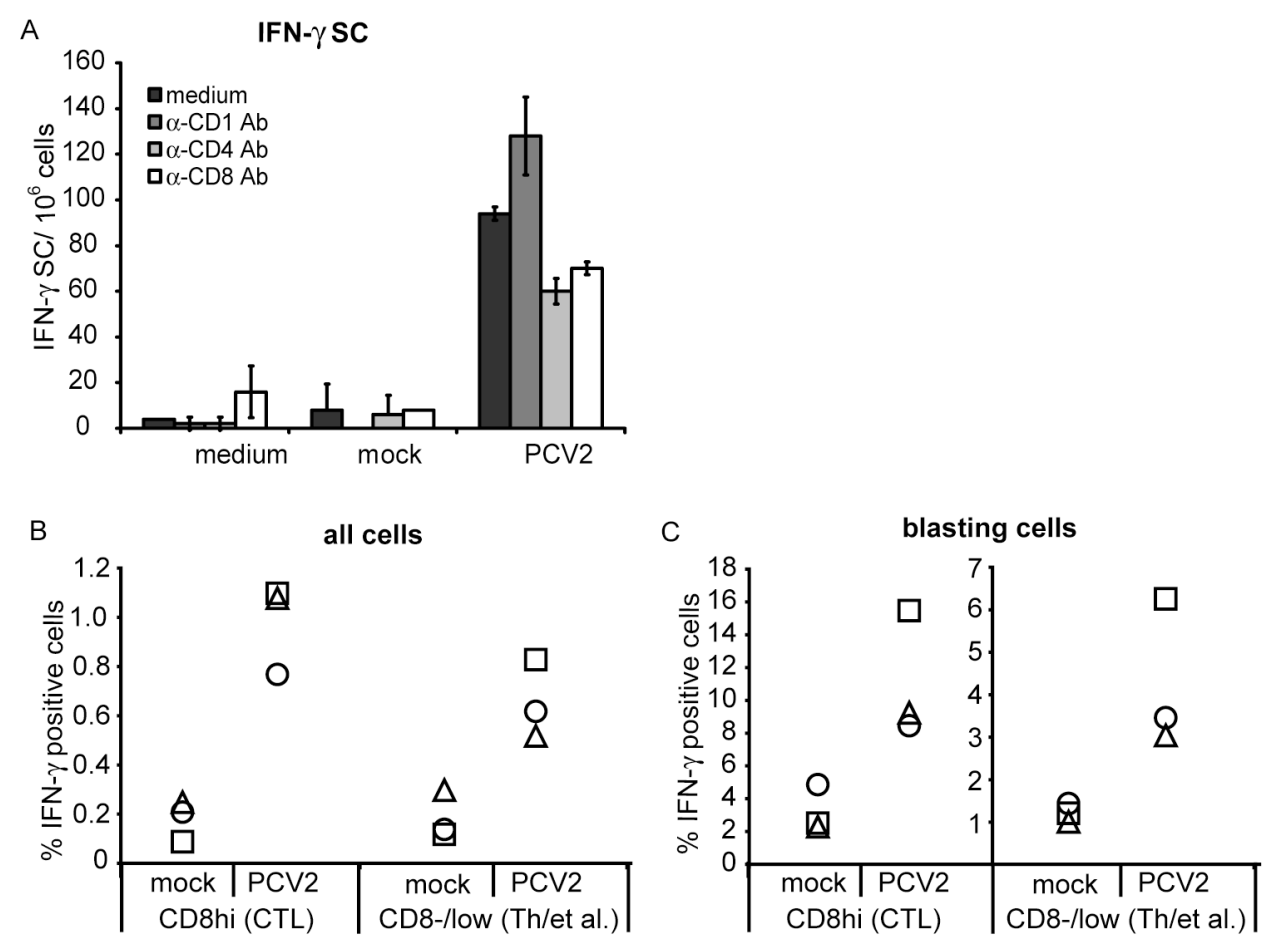
**Characterization of anti-PCV2 specific T lymphocytes**. (A) Anti-CD4 and -CD8 mAbs reduce IFN-γ SC. PBMC from PCV2-immune animals were treated with mAbs against the CD4 and CD8 T cell receptors, or anti-CD1 as control for 1 h prior to PCV2 or mock antigen re-stimulation for 5 days (as in Fig. 5). The IFN-γ SC were measured by ELISPOT assay, and calculated per 10^6 ^cells. Mean values of triplicates of one representative experiment +/- SD are shown. (B and C) IFN-γ SC detected by flow cytometry. PBMC from PCV2-infected piglets (3 months after infection) were re-stimulated with PCV2 or mock antigen for 5 days prior to staining for the presence of intracellular IFN-γ. The cells were also labelled for the surface markers CD8 to differentiate MHC class I restricted CTLs as CD8^hi ^cells from Th cells located within the CD8^-/lo ^cell population. The latter includes also NK cells and γ/δ T cells. Values shown on the y-axis represent the percentage of IFN-γ positive cells. The symbols represent the individual animals. In C the percentage of the IFN-γ positive cells within the blasting cells defined by gating on cells with high forward scatter is shown. For B and C, monocytic cells were excluded based on their CD172a expression.

When the *in vitro *PCV2 re-stimulation assays were repeated in the presence of anti-CD4 or anti-CD8 Ab, both treatments impaired the development of the IFN-γ SC (Fig. 6A). In contrast, anti-CD1 Ab did not decrease the number of IFN-γ SC induced by the PCV2 re-stimulation (Fig. 6A).

Considering the results obtained with anti-CD4 and anti-CD8 Ab in the ELISPOT assay, the PBMC from PCV2-immune animals, isolated 3 months after infection, were analysed further with respect to their phenotype by flow cytometry. Following *in vitro *re-stimulation, the IFN-γ SC were defined in terms of CD8 expression, and the presence of intracellular IFN-γ (Fig. 6B, C). MHC class I restricted CTLs were defined by gating on the CD8^hi ^population and the remaining lymphocytes by gating on the CD8^-/low ^cells. The later were composed of Th, NK cells, γ/δ T cells and B cells. Both CD8^hi ^CTLs and CD8^-/low ^cells were found to express the IFN-γ in response to PCV2 antigen (Fig. 6B). The IFN-γ expressing cells were clearly found to be reactive lymphocytes, represented by an increase in IFN-γ positive cells if the gate of interest was placed on blasting cells (Fig. 6C). The observation that cells isolated from naïve pigs showed no IFN-γ response following restimulation with PCV2 (data not shown) would imply that the observed IFN-γ production within the CD8^-/low ^cell population represented a Th cell-derived memory response. Together with the observed impairment of the PCV2-specific IFN-γ response by both anti-CD4 and anti-CD8 Ab (Fig. 6A) we conclude that PCV2-specific T lymphocytes reside within both CTL and Th populations, at least with respect to the IFN-γ response.

## Discussion

Although PCV2 will cause the development of diseases such as PMWS, not all PCV2-infected animals develop disease symptoms [10,27]; in fact the majority may remain asymptomatic. Moreover, a number of models, including the PCV2/PPV co-infection model in SPF piglets used for the present work, generate more often asymptomatic infections [28]. In view of the reports showing that PCV2 infection leads to modulation of T lymphocyte activity [23,24,29], the present work sought to characterize the T lymphocyte response in animals following infection with PCV2. This is particularly pertinent considering the reports on T lymphocytes in sow colostrum [30-32], and the reported transfer of antigen-specific T-cell immunity to piglets, either by transplacental passage or via the colostrum [30]. The study was designed to relate observations on the T lymphocyte subsets to the development of specific anti-PCV2 humoral immunity, since the presence of PCV2 specific neutralizing antibodies argues for the existence of PCV2 specific Th cells [16].

Accordingly, samples were employed from an experimental infection by PCV2 alone or in combination with PPV, in which the virus-inoculated animals remained asymptomatic. The asymptomatic outcome of PCV2/PPV dual infection in colostrum-fed piglets was also reported by other groups [10,28]. The exact reason that certain animals remain subclinically infected is not known but reflects the field scenario. The genetic background of the animals, environmental factors like stress and management factors like food change and bad hygiene have a certain impact on disease development [12]. Recently, certain PCV2 viral strains were correlated to different disease outcome [33,34]. Therefore, multiple reasons may have caused the failure of disease reproduction in this study. In general, the absence of clinical disease correlates with the presence of a PCV2 specific B and T cell response in the infected animals.

The experimental data from the present work is in agreement with published data on humoral immune defence development [28], in that the development of subclinical PCV2 infection was confirmed by the presence of PCV2-specific antibodies and PCV2 DNA in the blood. Interestingly, the PCV2 viraemia in this study was transient reflecting the ongoing PCV2 specific T and B cell immune response in the infected animals and clearance of the circulating virus. The pre-existence of PCV2 maternal antibodies can be ruled out, as the piglets tested at the age of 3 weeks and the mother sows displayed no PCV2 specific antibodies.

No differences in the levels of the PCV2 Ab titres were observed in the PCV2/PPV dual infected animals compared to the PCV2 single infected animals. When compared with the kinetics of the anti-PPV response, the development of the anti-PCV2 Ab appeared to be delayed, an observation also made by others [28]. This may reflect differences in the immunogenicity of the two viruses [35-38], but may also relate to the rate at which PCV2 replicates [21]. The response in the PCV2/PPV dual infected animals did precede that in the PCV2 single infected animals by 4 days. Although one could argue that the concomitant PPV infection may have activated the immune system to respond more rapidly against PCV2, further experimentation would be required to ascertain if such a short time delay has any significance. On the other hand, one has to consider the PPV infection that occurred accidentally at around day 10-14 p.i. in the PCV2 single infected animals and might have influenced the PCV2 specific Ab response of this group at later time points. Not to cause misinterpretation of the data, the PCV2 single infected group was only included in the statistical comparisons until day 10 p.i.

Considering the PCV2 infection, no apparent influence on the humoral response against PPV was observed: The titres and kinetics of the anti-PPV Ab response did not differ between the PPV single and PCV2/PPV dual infected groups.

A full appreciation of adaptive immune defences developing after PCV2 infection requires analysis of T lymphocyte activity, particularly when considering the influence of PCV2 on T lymphocyte numbers and activities [23,24,29]. The present work identified an influence on lymphocytes in asymptomatic animals, seen as a decrease in CD3^+ ^T cells and IgG^+ ^B cells in PCV2/PPV dual infected animals (relating to the published observations with symptomatic animals [23,24,29]). Being a transient decrease, followed by a significant rise in B and T cell numbers, the animals may recover from an immunosuppressed state caused by the concomitant PCV2/PPV infection. This recovery would explain the asymptomatic outcome.

Using ELISPOT with PBMC from PCV2-infected animals, we were able to estimate the development of the frequency of PCV2-specific T-cells. These were detected at an earlier time point (7 d p.i.) in the PCV2/PPV dual infected animals than in the PCV2 single infected animals. All PCV2-infected animals responded to PCV2 recall antigen stimulation with IFN-γ secretion by 21 days post-infection. This kinetics is slower in comparison to that reported for experimental infection of gnotobiotic pigs with human norovirus [39] - peaking at day 7 p.i., followed by a relative stability. Porcine reproductive and respiratory syndrome virus (PRRSV) also induces IFN-γ SC with a similar kinetics [40], the PRRSV-induced activity peaking at day 14 p.i., after which the numbers were relatively stable for the remainder of the experiment. Our data suggest that PCV2 is not a strong a T cell immunogen probably due to a slow replication of PCV2; the PCV2-specific humoral response is also slow to develop. Either of these scenarios would be in agreement with our previous demonstration that PCV2 alone does not activate professional antigen presenting cells or lymphocytes *in vitro *[19], and is seen to replicate slowly in primary cell cultures [21].

Early after infection, an unspecific IFN-γ response was observed in the PPV single infected animals: Similar levels of IFN-γ SC were induced by *in vitro *re-stimulation with the negative mock control antigen and PCV2 antigen. This response appears to be antigen-unspecific, possibly reflecting NK cells or γ/δ T cell activities. Interestingly, it was absent in the PCV2/PPV dual infected animals, indicating a possible immunomodulatory effect associated with the PCV2 infection. This relates to a recent publication showing that *in vitro *PCV2-infected PBMC displayed an impaired IFN-γ response upon recall stimulation with pseudorabies virus [29]. The reported effect was apparently mediated by IL-10, which can influence antigen-specific and antigen non-specific responses, as mediated by NK cells and γ/δ T cells.

The anti-PCV2 T lymphocyte response was further characterized using blocking antibodies which would interfere with functional CD4 and CD8 molecules. Analyses showed that the response involved both CD4^+ ^helper and CD8^+ ^cytotoxic T cells in the IFN-γ response. This was supported by an increase in IFN-γ secreting CD8^hi ^and CD8^-/low ^cells when PBMC from PCV2 infected animals were re-stimulated with PCV2 *in vitro*. Future studies are required to determine how such responses develop in animals with PCV2 associated diseases and after vaccination, and how they contribute to protective immunity.

## Conclusions

Asymptomatic PCV2-infected animals develop antigen-specific humoral and T cell responses, albeit with a relatively slow kinetics. The latter involves both CTL and helper T lymphocyte populations, confirmed as PCV2-specific through comparative stimulations with PCV2 and mock antigens.

## Methods

### Virus

Porcine circovirus type 2 (PCV2) pool 1452/3 and porcine parvovirus (PPV) pool 1005 were kindly provided by Francis McNeilly, QUB, Belfast. Virus titres were determined on the PCV2/PCV1-negative porcine PK15-A kidney cell line (also kindly provided by Francis McNeilly). Titres of 10^4.75 ^50% tissue culture infectivity dose (TCID_50_)/ml and 10^6.0 ^TCID_50_/0.5 ml were determined for the PCV2 and the PPV inocula respectively. In the ELISPOT assay, a lysate from PK15-A cells infected with the PCV2 strain Stoon 1010 [4] (also kindly provided by Francis McNeilly) was used.

### Experimental infection and clinical monitoring

The animal experiment was approved by the state committee for animal experimentation (authorization N° 18/04 delivered by the canton Bern) and carried out in accordance with the laws on care and use of laboratory animals in Switzerland.

Twenty 3-week old SPF piglets were randomly divided into four groups that were kept apart in separate isolated rooms of the institute BSL4 containment facility. The piglets received PCV2 (group 1), PPV (group 2), PCV2 + PPV (group 3), or Dulbecco's phosphate buffered saline (Invitrogen) (PBS) (group 4), administered intranasally on three consecutive days. At day 0, the piglets of the groups 1 and 3 were infected with PCV2 at a titre of 10^4.65 ^TCID_50_/animal; piglets of groups 2 and 3 were infected with PPV at a titre of 10^6.2 ^TCID_50_/animal. The piglets of the PBS control group (group 4) received corresponding volumes of PBS. On day 1 and 2 post infection (p.i.), additional doses of PCV2 and PPV were applied, at titres corresponding to 10^4.35 ^TCID_50_/animal and 10^5.9 ^TCID_50_/animal respectively. Body temperature and clinical symptoms were recorded daily, and the body weight every second to third day.

### Blood sampling

Blood was collected at day 0, 7 days (d), 10 d, 14 d, 17 d, 21 d, 24 d, 28 d and 35 d p.i., and diluted in Alsever's anticoagulant solution. By whole blood staining with Tuerk's solution (Dr. Grogg Chemie Ag, Bern, Switzerland), the total leukocyte number was calculated. Plasma samples were collected after a centrifugation step at 1,000 × g for 25 min and stored at -70°C. Peripheral blood mononuclear cells (PBMC) were isolated from the buffy coat fraction of blood by density centrifugation over Ficoll-Paque (1.077 g/liter) as described previously [41]. For long-term storage under liquid nitrogen, the PBMC were resuspended in Dulbecco's Modified Eagle's Medium (DMEM) containing 50% (v/v) FBS (all from Invitrogen) and 10% (v/v) DMSO.

### Anti-PPV and anti-PCV2 antibody ELISAs

Anti-PPV antibodies (Ab) were detected using a PPV competitive antibody ELISA kit Svanovir (10-7400-02; Svanova Biotech, Uppsala, Sweden). The percent inhibition (PI) values were calculated with the given formula: PI = 100-(MEAN OD samples/MEAN OD negative control), where OD = optical density as 450 nm. Positive plasma samples showed a PI value greater than 50 and samples exceeding a PI value of 80 were considered as strongly positive.

Anti-PCV2 specific Ab were detected with an indirect anti-PCV2 Ab ELISA, established in our laboratory. Briefly, Nunc MaxiSorb™ plates were coated with 1 μg/ml of PCV2 ORF2 virus-like particles (VLPs) (kindly provided by Dr. C. Andreoni, Immunology, Discovery Research, Merial, Lyon, France) in 100 μl PBS overnight (O/N). The plates were blocked with PBS/1% (w/v) dried skimmed milk for 1 h at 37°C, and washed once with PBS/0.05%(v/v) Tween-20 (washing buffer). The plasma samples were added in dilutions from 1:50 to 1:800 in PBS/1% (w/v) bovine serum albumin (BSA, bovuminar cohn fraction V, Intergen Company Serologicals) for 1 hour (h) at room temperature (RT). The plates were washed five times and horseradish peroxidase-conjugated polyclonal anti-swine IgG F(ab')_2 _fragments (Jackson ImmunoResearch), diluted in PBS, 0.05% (v/v) Tween-20, 1% (w/v) dried skimmed milk, 1% (v/v) rabbit serum (Sigma) was added. After 1 h incubation at 37°C and five washing steps, the substrate O-Phenylenediamine (OPD) (Sigma: P-8787) plus 0.0001% (v/v) H_2_O_2 _was added for 40 minutes, and the absorbance measured at 450 nm. Samples were considered as positive if the optical density (OD_450 nm_) values equalled or exceeded 0.1 at dilutions higher than 1:100. Samples from animals treated with PBS were always below OD_450 nm _0.1.

### Restimulation of PCV2 specific cells

For *ex vivo *IFN-γ ELISPOT assays, freshly isolated PBMC were resuspended in DMEM, 10% (v/v) FBS, 1× Penicillin/Streptomycin, 20 μM 2-mercaptoethanol (2-ME) (all from Invitrogen). 200 μl of 2.5 × 10^6 ^PBMC/ml were seeded in 96-well multiscreen plates, and the cells restimulated with PCV2 virus lysate of the strain Stoon 1010 [4] or with mock lysate, diluted to give an MOI of 1 TCID_50_/cell (or equivalent dilution for the mock) for 24 h.

Thawed PBMCs were also employed for the IFN-γ ELISPOT. They were seeded at a concentration of 3 × 10^6 ^cells/ml in 24 well plates, and restimulated with PCV2 lysate or mock lysate at an MOI of 0.1 TCID_50_/cell (or equivalent dilution) for 5 days. The cells were harvested, counted, and stained for flow cytometry analysis or re-plated on 96-well mutliscreen plates at a concentration of 1.25 × 10^6^cells/ml for IFN-γ ELISPOT analysis. When indicated in the results, 50 U/ml of the recombinant porcine cytokine IL-2 (R&D systems) were added to the cultures.

In order to block the CD4 or CD8 dependent IFN-γ secretion, 50 μl of anti-CD4 Ab (74-12-4), anti-CD8 Ab (11/295/33), or control anti-CD1 Ab (76-7-4) were incubated with the cells for 1 h at 37°C, prior to PCV2 restimulation [25].

### IFN-γ ELISPOT assay

The ELISPOT was performed with minor modifications as described previously [42]. Briefly, 96-well multiscreen plates (Millipore MAIPS4510) were coated with 0.5 μg/ml of mouse anti-porcine IFN-γ mAb (P2G10, BD bioscience, Allschwil, Switzerland) diluted in PBS at 4°C O/N. The plates were washed twice with 200 μl of cold DMEM, 10% (v/v) FBS and blocked for 2 h at 37°C. PBMC were seeded at the above mentioned concentrations into the plates, and incubated at 39°C. Reference positive control cells were activated with 10 μg/ml of the mitogen ConA (Amersham Pharma Biotech, Uppsala, Sweden). After 24 h, the medium was aspirated and the cells lysed by addition of cold distilled water for 5 minutes. After washing three times with PBS, 0.5% (v/v) Tween-20 (washing buffer), the plates were incubated with 0.17 μg/ml biotinylated anti-porcine IFN-γ Ab (P2C11, BD Biosciences, Allschwil, Switzerland) in PBS at 4°C O/N. The plates were washed five times in washing buffer, and incubated with Streptavdin HRP (DakoCyomation, Dako AG, Baar, Switzerland) diluted in PBS 1 h at 37°C. After an additional washing step, the plates were revealed with SIGMAFAST™ 3,3'Diaminobenzidine tablets (Sigma, Buchs, Switzerland) dissolved in distilled water. After 20 min incubation at RT in the dark and drying of the plates, the number of spots was determined with a computer-assisted video image analyzer (ELISPOT reader, AID, GmbH, Strassberg, Germany).

### Antibodies

For phenotyping of cells, the following Ab were used: fluorescein (FITC) conjugated goat anti swine IgG (H+L)(Jackson ImmunoResearch, Milan Analytica, Magden, Switzerland); mouse anti-swine CD8 beta chain (PG164A, Veterinary Medical Research & Development, Inc., Pullman, WA); mouse anti-swine IFN gamma (IFN-γ) (P2G10 BD biosciences, Allschwil, Switzerland); anti-swine workshop cluster 3a (SWC3a; 74-12-15A); mouse anti-swine CD8 (11/295/33); mouse anti-swine CD3a (PTT3/FyH2). The latter three monoclonal Ab (mAb) were produced from hybridomas kindly provided by Dr. Armin Saalmüller, University of Veterinary Medicine, Vienna, Austria. Anti-Capsid protein ORF2 mAb 7G5-G4-A1 (IgG2a) was kindly provided by Dr. Allan Gordon QUB, Belfast.

### Flow cytometry

The PBMC were stained with anti-CD3a and anti-IgG (H+L) Ab to analyse the relative levels of T- and B-lymphocytes respectively. The absolute numbers of lymphocytes were calculated relative to the total leukocyte numbers. The PBMC were stained with anti-SWC3a, anti-CD4 and after a fixation and permeabilisation step (Fix and Perm kit, ADG An Der Grub, Bio research GMBH, Austria) with anti-Capsid Ab to analyse the presence of Capsid antigen in the monocytic cells.

For intracellular IFN-γ detection, PCV2 re-stimulated PBMC were treated with 2 μg/ml of Brefeldin A (Sigma, Buchs, Switzerland) 4 h prior to staining with anti-SWC3a, anti-CD8 and anti-IFN-γ Ab. The cells were fixed and permeabilised with the Fix and Perm kit (ADG An Der Grub, Bio research GMBH, Austria) before staining for the intracellular cytokine IFN-γ. Antibodies were detected by fluorescein, phycoerythrin or biotin-conjugated goat F(ab')_2 _anti-mouse isotype specific immunoglobulins (Southern Biotechnology Associates, Bioconcept, Switzerland). Streptavidin RPE-Cy5 (DakoCyomation, Dako AG, Baar, Switzerland) was used to develop the biotinylated conjugates.

### Statistical analysis

The statistical significance of the differences between groups was analysed with SigmaStat version 3.0, employing ANOVA on ranks and t-tests. The PCV2 single infected group was included in the statistical analysis until 10 d p.i. but excluded from the analysis of later time points due to PPV contamination.

## Authors' contributions

ES: performing the animal experiment, ELISA, ELISPOT assay, restimulation assays, and FACS analysis, analysing the data, and writing the manuscript. CB: overviewing and discussing the data, developing the ex vivo ELISPOT assay and performing some of the assays, developing the in-house ELISA and restimulation assays, and drafting/revising the manuscript. HG: help with isolating the PBMC, and their long term storage. AS: intellectual contribution to planning the animal experiment and interpretation of the data, assisting with the animal experiment, and revising the manuscript. KMC: senior author responsible for overseeing the planning of the experimental work and the interpretation of the data, discussing the data and overviewing its interpretation, reviewing and revising the manuscript. All authors read and approved the final manuscript.
